# The Impact of the Magnitude of the Group of Bullies on Health-Related Quality of Life and Academic Performance Among Adolescents

**DOI:** 10.1007/s10578-021-01290-8

**Published:** 2021-12-01

**Authors:** Ángela de Lourdes Martín-Pérez, Juan José Gascón-Cánovas

**Affiliations:** 1 Department of Psychiatry, Santa Lucía Hospital, Cartagena, Spain; 2 Department of Preventive Medicine and Public Health, Faculty of Medicine, University of Murcia, Campus de Espinardo, 30110 Murcia, Spain

**Keywords:** Victimization, Adolescent, Health-related Quality of Life, Academic performance

## Abstract

This study examines the consequences that physical and verbal/social victimization by peers and the magnitude of the groups of bullies have on academic performance and the psychological and social domains of Health-related Quality of Life (HRQoL). 1428 secondary school students in the south-east Spain completed the Spanish version of the “Adolescent Peer Relations Instrument-Bullying” and “KIDSCREEN-52” questionnaires in order to analyse, respectively, peer victimization and the psychological and social domains of HRQoL. Data on sociodemographic characteristics and academic achievement was also collected. Findings emphasise the potential of peer victimization in all its forms as risk factors explaining poor HRQoL in psychological, social and emotional domains. The number of bullies was an imponent and significant risk factor that explains a worse HRQoL in the five socio-psychological dimensions studied (Odds Ratio 4.08, Odds Ratio 9.25, Odds Ratio 4.69, Odds Ratio 2.91, Odds Ratio 11.92). Nevertheless, peer victimization rarely seems to affect academic achievement. Results suggest that much of prevention and intervention are still needed to reduce peer victimization, focusing on large bullies’ groups and their harmful impact on adolescent’s HRQoL.

## Introduction

Unfortunately, peer victimization is a societal phenomenon that has become increasingly common and problematic [1], above all in adolescence [2]. At this stage of life, the number of individuals who are the object of aggressive and/or unsolicited behaviour from peers in any of its forms (physical, verbal or social victimization) is considerable [3]. In fact, approximately 10–30% of adolescents in our planet admit that they have been involved in this type of violence, either as victims or bullies. Furthermore, there is a heterogeneous distribution of this behaviour among different countries [4]. Specifically, in Spain, the probability of having been harassed by colleagues is above the European Union average and has increased from 17.1% in 2006 to 21.5% in 2014 [5]. More recent studies have shown even higher rates of peer victimization during the last years, suggesting a prevalence of 72% among the adolescent Spanish population [6]. In other words, despite all efforts put into studying and preventing peer victimization, it is still a worrying matter increasing worldwide and, specifically, in Spain. Previous authors have already highlighted the importance of prevention strategies against peer victimization [7]. However, most of them focus on the US or Scandinavian countries. Therefore, new research is needed to analyse other population groups such as the present study, which examines the Spanish adolescent population.

Cyberbullying—another variety of peer victimization– has recently appeared, caused by the incorporation of emerging technologies into our digital society [8]. However, from all types of violence, social and verbal harassment continue to be by far the most common forms of victimization [9]. Specifically, in Spain, recent studies have shown prevalence rates of social and verbal victimization of 46.1% and 29.5%, respectively, of the total types of violence suffered by adolescents [10].

Several previous studies have analysed the effects of peer victimization on adolescents’ health and highlighted its significant negative impact on both physical [11] and mental health [8, 12], especially, when different types of harassment are combined [13]. Health concept has been defined by The World Health Organization as not only the absence of physical or mental illness, but “a state of complete physical, mental and social well-being” [14]. In connection with this, the term of “Health-Related Quality of Life (HRQoL)” has been commonly used as a multidimensional construct that covers physical, mental and social functioning [15]. In this sense, it has been clearly demonstrated that adolescents who are victimized by their peers have a poorer HRQoL than those who are not [16]. In addition, this not only affects adolescence but also usually continues during adulthood, even if violence episodes have stopped [17]. To date, most studies on this topic have focused on studying the negative effects of victimization on overall HRQoL [18, 19]. However, less is known about the outcome of victimization on each specific HRQoL domain. It would be interesting to know which domains of HRQoL are most affected in order to improve the efforts made towards personal recovery of victimized adolescents. The aim of this study is to investigate how different types of peer victimization affect psychological and social domains of adolescents’ HRQoL.

The effects of peer victimization on victims’ academic performance are controversial as some studies indicate that they are clearly harmful [20], while others indicate that the impact is negligible or even null [21, 22]. Even less known is the impact on academic performance depending on what type of victimization the adolescent has suffered. This study aims to contribute by providing further data that may help shedding light on this controversial issue and adding new information about the impact that different types of victimization may have on academic achievement.

Although the consequences of peer victimization on adolescent population have been commonly studied, less attention has been paid to the impact that the group of bullies may have on adolescents' HRQoL or academic performance. The present study is one of the first to explore the importance of the magnitude of the group of bullies as a possible risk factor that may explain a further worsening of the victimized adolescents’ HRQoL or academic performance.

Accordingly, this study was conducted to assess the consequences of different forms of peer victimization (physical and verbal/social) on the psychological and social domains of HRQoL. This study aims to provide further data that may clarify the impact of peer victimization on academic performance. We further sought to explore whether the magnitude of the group of bullies is a risk factor that by itself could explain a worsening of victims' HRQoL and academic performance.

## Methods

### General Design and Participants

A retrospective cross-sectional study was conducted. The study included all secondary school students (12–16 years old) from all schools in the town of Torre Pacheco (South-east Spain) (N = 1476). This area was chosen due to its great ethnic variety.

Information was collected during the last 30 days of the school year from self-completed questionnaires. The questionnaires were administered by major teachers during class time. Adolescents were given 1 h to complete the entire questionnaire. All questionnaires were completed anonymously after written consent was obtained from parents via Parents’ Associations in all participating schools.

### Measures

Sociodemographic variables were collected by means of a questionnaire designed ad hoc. Those variables include age, sex, family structure (*Nuclear*, *Mononuclear* or *No parents at home*), ethnic origin (parents’ birthplace: *both Spanish*, *one Spanish*, *Maghreb*, *Latin-Ecuador* or *Others* which included all other options) and parental educational attainment**.** Based on the procedure proposed by the Spanish Society of Epidemiology [23], information about social class in terms of parents’ employment was also included. For both social class and parental educational attainment, the highest positions of both parents were taken as a reference.

Peer victimization was measured using an adapted-to-Spanish version of the validated Adolescent Peer Relations Instrument-Bullying (APRI), developed by Parada [24, 25]. This scale contains 18 items and measures two dimensions of peer victimization: physical victimization (6 items) and verbal/social victimization (12 items). Each item is rated on a 4-point Likert scale indicating the frequency of victimization from the beginning to the end of the academic year (nine months) (0 = *Never/seldom,* 1 = *Frequently,* 2 = *Very often,* 3 = *Constantly).* The score for each dimension was calculated as the sum of the respective items. The higher the score on each subscale, the stronger the victimization suffered by the teenager. To facilitate the interpretation of this study, verbal/social victimization was categorized into tertiles, and physical victimization was divided into four distinct categories according to the number of physical violence episodes suffered by the adolescent (0, 1, 2–4, ≥ 5). The reliability of this sample was 0.85 and 0.92 for the Cronbach’s Alfa for “physical victimization” and “verbal/social victimization”, respectively. In addition to its good internal consistency, this questionnaire was selected for its applicability to the adolescent population and for its ability to differentiate between different subtypes of victimization.

The magnitude of the group of bullies was assessed by calculating the total number of peers who had bullied the adolescent during the last academic year (0 = *None*, 1 = *1 or 2 bullies*, 2 = *3 bullies*, 3 = *4 or more bullies*).

Health-related Quality of Life (HRQoL) was measured by analysing five out of ten domains of the validated and adapted to Spanish KIDSCREEN‐52 questionnaire [26]: *Psychological Well-being* (6 items), *Mood and Emotions* (7 items), *Social Support and Peers* (6 items), *School Environment* (6 items) and *Social Acceptance* (3 items). Each item is rated on a five-point Likert scale corresponding to feelings of well-being over the previous week (1 = *Never*, 2 = *Seldom*, 3 = *Sometimes*, 4 = *Often*, 5 = *Always*). Rates were calculated independently for each dimension as T-values of the Rasch scores corresponding to the sum of the response options [27]. Higher scores correspond to a better quality of life. The internal consistency of the five KIDSCREEN-52 domains analysed in the study ranged between a Cronbach's alpha of 0.78 and 0.85. This questionnaire was selected because of its applicability in different cultural contexts and its practical use as well as its good internal consistency.

Academic performance was estimated using two indicators: *Academic excellence* and *Academic failure* in core secondary school subjects (Mathematics, Social Sciences, English Language, Natural Sciences and Spanish Language). *Academic excellence* was assessed by calculating the average of the scores obtained in these six subjects in a range from 0 to 4 *(Poor or below F* = *0, Not acceptable F* = *1, Pass C* = *2, Very good B* = *3, Excellent A* = *4)*. For this purpose, normalized values were used by calculating the average school score for each academic subject. The adolescent was considered to have achieved academic excellence when his or her grade point average was above one standard deviation from his/her school average. *Academic failure* was derived by determining the number of subjects with a grade lower than a pass mark. The adolescent was considered to have failed academically if he/she had one subject below a pass mark. Data was obtained from the information provided by the students themselves regarding the last exam they had taken.

### Statistical Analysis

Adolescents’ sociodemographic characteristics were collected using descriptive analysis by calculating frequencies and percentages.

Several multivariate analyses were performed using binary logistic regression to estimate peer victimization (physical and social/verbal) and “bullies’ group density” associations with the likelihood of a poor HRQoL, academic failure or not achieving academic excellence. Each form of peer victimization and “bullies’ group density” variable was taken as an independent variable, and HRQoL and academic performance as criterion variables. All models were adjusted by sociodemographic variables that had previously had p-values below 0.10 in the univariate logistic model.

For KIDSCREEN-52, the mean scores varied around 50 (SD = 10) due to T-value standardization [27]. Poor HRQoL was assigned to values below the 50th percentile (P50) or Median. Dichotomized values were used for dependent variables in logistic regression models: KIDSCREEN-52 dimensions (T-values below the 50th percentile were represented by a score of 1, and all others by zero); degree of academic excellence (1 = average score equal to or greater than one standard deviation from school average, 0 = average score less than one standard deviation from school average); degree of academic failure (1 = one or more failures, 0 = no failure).

When half or more of the subscale items were available within each APRI domain and KIDSCREEN scale for a particular participant, missing data was replaced by the mean score of the remaining items on the same subscale. Otherwise, data was excluded for the analysis of the affected dimension [28]. The extent of missing data for each APRI and KIDSCREEN-52 domain ranged from 16 missing data for the "Physical Victimization" scale to 90 for the "School Environment" scale.

All analyses were performed using the Statistical Package for Social Sciences SPSS—24.0. p values < 0.05 were considered to be statistically significant.

## Results

### Sociodemographic Characteristics

The participation rate reached 96.7% (n = 1428). Fifty-four adolescents did not complete the questionnaire, thus an effective rate of 95.6% was achieved (n = 1411). Participants included 745 boys (52.8%) with a mean age of 14.8 (SD 1.4) and an age range of 12–18. The majority (85.3%) came from nuclear families, in which both parents were Spanish (64.5%). Adolescents from Maghrebi (18.2%) or Latin backgrounds (9.6%) were the next two most common categories in frequency. Two-thirds of the main breadwinners worked in semi-skilled or unskilled manual jobs and only one fifth had higher education (Table 1).

**Table 1:** Characteristics of the studied sample (n = 1411)

	n	(%)
Gender		
Female	666	(47.2)
Male	745	(52.8)
Age range (years)		
[12.0–13.9]	488	(34.6)
[14.0–15.9]	649	(46.0)
[16.0–18.9]	274	(19.4)
Type of family		
Nuclear	1203	(85.3)
Mononuclear	193	(13.7)
No parents at home	15	(1.1)
Parental ethnic origin		
Both Spanish	910	(64.5)
One Spanish	39	(2.8)
Maghreb	257	(18.2)
Latin-Ecuador	135	(9.6)
Other	70	(4.9)
Social class^a^		
I or II	278	(19.7)
III	140	(9.9)
IV or V	883	(62.6)
VI	110	(7.8)
Parental educational attainment		
No education/primary education	456	(32.3)
Secondary education	663	(47.0)
Higher education	292	(20.6)

^a^Social class; I. Higher managerial; II. Intermediate managerial; III. Supervisory, junior managerial; IV. Skilled manual occupations; V. Unskilled manual occupations; VI. Unemployed/pensioner/retiree

### Health-Related Quality of Life According to Sociodemographic Characteristics

After controlling for potential confounders (Table 2), multivariate analyses showed that girls had a lower probability of having a problematic HRQoL in the following dimensions: *School Environment* (CI 95% OR 0.51–0.85) and *Social Acceptance* (OR 0.73, p = 0.019), but conversely had a greater risk of poorer *Mood and Emotions* quality of life values (OR 1.49, p = 0.005).

**Table 2:** Poor HRQoL^a^ according peer victimization

	“PWB” < P50	“MAE” < P50	“SSAP” < P50	“SE” < P50	“SA” < P50
	OR_adj_ (CI 95%)	OR_adj_ (CI 95%)	OR_adj_ (CI 95%)	OR_adj_ (CI 95%)	OR_adj_ (CI 95%)
Gender					
Female vs male	1.28(0.99–1.64)	1.49(1.13–1.96)*	–	0.66(0.51–0.85)**	0.73(0.56–0.95)*
Age range (years)					
[12.0–13.9]	1.0	1.0	1.0	1.0	1.0
[14.0–15.9]	1.89(1.40–2.54)**	1.28(0.93–1.77)	1.40(1.03–1.92)*	1.68(1.26–2.25)**	–
[16.0–18.9]	1.76(1.22–2.53)*	1.45(0.99–2.12)	1.22(0.82–1.80)	1.38(0.96–2.00)	–
Type of family					
Nuclear	1.0	1.0	1.0	1.0	1.0
Mononuclear	–	1.20(0.81–1.77)	–	1.39(0.98–1.96)	–
No parents at home	–	1.48(0.44–4.97)	–	0.45(0.10–2.02)	–
Parental ethnic origin					
Both Spanish	1.0	1.0	1.0	1.0	1.0
One Spanish	1.46(0.71–3.09)	1.23(0.52–2.90)	1.22(0.52–2.86)	0.86(0.38–1.94)	1.34(0.62–2.90)
Maghreb	1.49(1.07–2.09)*	1.37(0.95–1.97)	2.08(1.46–2.98)**	0.66(0.45–0.95)*	1.17(0.82–1.68)
Latin-Ecuador	1.14(0.73–1.76)	1.42(0.90–2.24)	1.73(1.11–2.69)*	0.96(0.63–1.48)	1.05(0.66–1.66)
Other	1.06(0.58–1.96)	2.82(1.60–4.94)**	1.81(1.01–3.28)*	1.40(0.82–2.40)	1.27(0.70–2.31)
Social class^b^					
I or II	1.0	1.0	1.0	1.0	1.0
III	0.63(0.38–1.05)	1.33(0.76–2.32)	1.08(0.61–1.90)	–	0.92(0.54–1.58)
IV or V	0.80(0.58–1.12)	1.38(0.92–2.06)	1.21(0.79–1.85)	–	1.09(0.76–1.56)
VI	1.15(0.69–1.93)	2.17(1.22–3.86)*	1.38(0.75–2.53)	–	1.60(0.93–2.75)
Parental educational attainment					
No education/primary education	1.0	1.0	1.0	1.0	1.0
Secondary education	–	–	0.97(0.71–1.33)	–	–
Higher education	–	–	0.93(0.60–1.45)	–	–
Physical victimization					
0	1.0	1.0	1.0	1.0	1.0
1	0.96(0.66–1.38)	1.22(0.81–1.84)	1.08(0.73–1.60)	0.86(0.59–1.24)	2.31(1.58–3.38)**
2–4	1.68(1.18–2.39)*	2.49(1.71–3.62)**	1.91(1.32–2.75)**	1.39(0.98–1.97)	5.07(3.55–7.25)**
≥ 5	3.77(2.36–6.03)**	8.35(5.10–13.68)**	3.14(1.95–5–06)**	2.24(1.40–3.61)**	15.86(9.60–26.20)**
Verbal/social victimization					
1st Tertile (< 1)	1.0	1.0	1.0	1.0	1.0
2nd Tertile [1–3]	1.73(1.24–2.42)**	2.11(1.40–3.18)**	1.94(1.34–2.82)**	1.22(0.88–1.69)	1.61(1.02–2.54)*
3rd Tertile [4–52]	2.84(2.08–3.88)**	5.48(3.78–7.95)**	3.20(2.27–4.52)**	1.63(1.21–2.20)**	9.06(6.19–13.24)**
Group of bullies					
None	1.0	1.0	1.0	1.0	1.0
1–2 bullies	1.70(1.25–2.30)**	2.22(1.58–3.12)**	1.18(0.84–1.67)	1.10(0.80–1.51)	3.11(2.25–4.28)**
3 bullies	2.59(1.50–4.46)**	5.50(3.16–9.57)**	2.81(1.61–3.89)**	2.25(1.30–3.89)*	8.73(5.11–14.91)**
≥ 4 bullies	4.08(2.47–6.74)**	9.25(5.45–15.70)**	4.69(2.86–7.70)**	2.91(1.77–4.78)**	11.92(7.11–20.00)**

*OR*_*adj*_ odds ratio adjusted by sociodemographic characteristics that previously had shown p values below 0.10 in the univariate logistic model, *CI* confidence interval; HRQoL dimensions: *PWB* Psychological well-being, *MAE* mood and emotions, *SSAP* social support and peers, *SE* school environment, *SA* social acceptance

*p < 0.05; **p < 0.001

^a^Values below the median (< 50th percentile)

^b^Social class: I: Higher managerial; II: Intermediate managerial; III: Supervisory, junior managerial; IV: Skilled manual occupations; V: Unskilled manual occupations; VI: Unemployed/pensioner/retiree

The youngest adolescents were less likely to have lower scores in most HRQoL categories, especially when compared to the middle age group in *Psychological Well-being* (OR 0.52, p < 0.001), *Social Support and Peers* (OR 0.71, p = 0.034) and *School Environment* values (OR 0.59, p < 0.001), and when compared to the oldest group in *Psychological Well-being* (OR 0.57, p = 0.002).

Social class had a significant effect only in the case of *Moods and Emotions* quality of life values. Adolescents from lower classes had 2.17 times more risk of having worse HRQoL than adolescents from classes I and II (p = 0.009).

Compared to adolescents who have two Spanish parents, children of Maghreb, Latin or other ethnicities had 2.08, 1.73 and 1.81 times more risk, respectively, of having worse scores in the *Social Support and Peers* subscale. However, Maghrebi ethnicity turned out to be an independent protective factor in the HRQoL related to *School Environment* when compared to adolescents who have two Spanish parents (CI 95% OR 0.45–0.95).

The type of family and parental educational attainment covariates were not significant in any of the regression analyses, thereby indicating that they have no influence on adolescent HRQoL.

### Academic Performance According to Sociodemographic Characteristics

The adjusted analyses (Table 3) showed that boys, older students and those who came from non-nuclear families obtained worse results for academic performance. Also, academic achievement worsened significantly in the least privileged classes.

**Table 3:** Poor academic performance and peer victimization

	Academic excellence	Academic failure
	OR_adj_ (CI 95%)	OR_adj_ (CI 95%)
Gender		
Female vs male	2.20(1.54–3.15)**	–
Age range (years)		
[12.0–13.9]	1.0	1.0
[14.0–15.9]	1.18(0.81–1.31)	1.29(0.97–1.71)
[16.0–18.0]	0.32(0.16–0.64)**	1.80(1.24–2.56)*
Type of family		
Nuclear	1.0	1.0
Mononuclear	0.38(0.18–0.77)*	2.09(1.41–3.09)**
No parents at home	4.27(1.04–17.50)*	0.67(0.14–3.19)
Parental ethnic origin		
Both Spanish	1.0	1.0
One Spanish	1.06(0.41–2.77)	0.81(0.37–1.75)
Maghreb	0.71(0.40–1.28)	1.77(1.22–2.56)*
Latin-Ecuador	0.16(0.05–0.53)*	2.07(1.27–3.39)*
Other	0.54(0.21–1.42)	1.51(0.84–2.74)
Social class^a^		
I or II	1.0	1.0
III	0.91(0.51–1.63)	1.46(0.91–2.35)
IV or V	0.58(0.36–0.92)*	1.92(1.35–2.73)**
VI	0.40(0.14–1.09)	1.66(0.94–2.94)
Parental educational attainment		
No education/primary education	1.0	1.0
Secondary education	1.73(1.09–2.73)*	0.76(0.57–1.02)
Higher education	2.17(1.23–3.83)*	0.56(0.38–0.84)*
Physical victimization		
0	1.0	1.0
1	0.99(0.62–1.57)	1.03(0.74–1.43)
2–4	0.89(0.54–1.47)	0.95(0.67–1.34)
≥ 5	0.70(0.31–1.61)	1.67(1.01–2.77)*
Verbal/social victimization		
1st Tertile (< 1)	1.0	1.0
2nd Tertile [1–3]	0.84(0.55–1.28)	1.13(0.83–1.52)
3rd Tertile [4–52]	0.70(0.47–1.01)	1.17(0.88–1.56)
Group of bullies		
None	1.0	1.0
1–2 bullies	0.83(0.55–1.26)	0.96(0.72–1.28)
3 bullies	0.58(0.24–1.43)	1.15(0.66–2.02)
≥ 4 bullies	0.10(0.01–0.71)*	1.11(0.65–1.88)

*OR*_*adj*_ odds ratio adjusted by sociodemographic characteristics that previously had shown p values below 0.10 in the univariate logistic model, *CI* confidence interval

*p < 0.05; **p < 0.001

^a^Social class: I: Higher managerial; II: Intermediate managerial; III: Supervisory, junior managerial; IV: Skilled manual occupations; V: Unskilled manual occupations; VI: Unemployed/pensioner/retiree

By contrast, good parental educational attainment was considered as an independent protective factor and adolescents whose parents had secondary or higher education were more likely to achieve academic excellence (OR 1.73 with p = 0.02, and 2.17 with p = 0.007, respectively); likewise, children whose parents had enjoyed higher education were also less likely to fail academically (CI 95% OR 0.38–0.84).

Compared to adolescents who have two Spanish parents, teenagers from Latin background had significantly worse academic performance, with a two-fold increase in the risk of academic failure (CI 95% OR 1.26–3.38) or not achieving *Academic excellence* (CI 95% OR 0.05–0.53). Results also revealed that Maghrebi children were most likely to fail (OR 1.77, p = 0.003).

### Associations of HRQoL with Peer Victimization and the Magnitude of the Group of Bullies

The magnitude of the group of bullies was a significant risk factor explaining lower HRQoL in all KIDSCREEN-52 subscales. Furthermore, both types of analysed victimization (physical, verbal/social) were closely associated with worse scores in all the HRQoL subscales. The most affected dimension by all types of victimization was *Social Acceptance*, followed by *Moods and Emotions* which, in the most serious cases of harassment, had a 15.86 and 8.35 (p < 0.001) increased risk, respectively, of worse scores in the event of physical violence. In the case of suffering verbal/social violence, an increase in risk of 9.06 and 5.48 (p < 0.001), respectively, was detected in these two dimensions (Table 2).

### The Role of Peer Victimization and the Magnitude of the Group of Bullies in Academic Performance

Multivariate analyses (Table 3) revealed that only serious physical victimization (score ≥ 5 on APRI scale) had an academic impact on adolescents, as physically harassed children were 1.67 times more likely to fail academically (p = 0.048). The density of the group of bullies had an independent effect on poor academic results: there was a 10 times greater risk of not achieving academic excellence when there were more than four bullies (CI 95% OR 0.01–0.71, p = 0.022).

## Discussion

The results of this study show that adolescents victims of peer victimization—in any of its manifestations (physical and verbal/social)—have poorer Health-related Quality of Life in psychological, emotional and social domains. Furthermore, the size of the group of bullies, an as-yet poorly studied phenomenon, clearly has a significant independent negative impact on HRQoL.

Previous research has shown that peer victimization has a harmful effect on the overall HRQoL of adolescents suffering from this type of harassment [16, 18, 19]. This investigation demonstrates how it is maintained when studying the influence of peer victimization on specific HRQoL socio-psychological domains, with physical victimization being the most damaging. Previous research has reported that physical victimization is the most harmful [29] while others suggested that social victimization is the one that most affects adolescents’ psychological wellbeing [30]. This study reveals physical victimization as the one that most damages adolescents’ HRQoL. One hypothesis that could explain this finding is that, usually, indirect victimization behaviours (e.g., social exclusion, etc.) are regarded as less serious than physical ones. It may occur because the lack of visible physical injuries could result in a lack of awareness of the seriousness of the behaviours and, therefore, a less damaging impact on adolescents’ HRQoL [29]. On the other hand, it could be explained by the fact that young people who have experienced physical victimization are more likely to have also experienced other violent behaviours (e.g., insults while being assaulted); whereas those who have suffered verbal or social victimization may not have suffered physical abuse. Previous studies have shown that experiencing multiple types of victimization has worse HRQoL outcomes [11]. Therefore, those who have experienced physical victimization along with other violent behaviours would have a greater impact on their HRQoL. In addition, as other studies have shown, this research reaffirms that the more serious and frequent the violence episodes are, the more pronounced the impact on adolescents’ HRQoL is [13, 31].

Results show how HRQoL’s psychological domains are severely affected in adolescents who have experienced both physical and verbal/social victimization, in fact, one of the most affected dimensions in the study is “*Mood and Emotions*”. These findings are in line with those studies suggesting that young people who have been bullied by their peers have higher rates of psychological distress [32]. It is well known that emotional/psychological distress is associated with a higher likelihood of poorer mental health [33]. The importance of this data lies in the fact that peer victimization is most frequent at secondary schools [34] and occurs at a critical age in terms of the onset of mental disorders [35], which is one of the most important causes of Disability Adjusted Life Years lost in young people [36]. The results of this study suggest that victims’ interventions should focus on strengthening the psychological and emotional spheres of HRQoL. Also, efforts to prevent peer victimization should be concentrated on this period of life, since these mentioned devastating consequences can extend into adulthood [17].

The finding that the magnitude of the group of bullies acts as an independent negative factor that affects HRQoL is especially noteworthy, since it is yet an unstudied phenomenon. These results are logical and expected, as one may feel more unprotected and less socially supported the more bullies are harassing oneself. These findings also support that empowering adolescents not to imitate or follow bullies is a way to avoid the creation of big groups of bullies and, thus, prevent worse future consequences on adolescents' HRQoL. On the other hand, it has also been demonstrated that suffering peer victimization is a risk factor to become a bully [37]. People who have suffered victimization and have become bullies are known as “bully-victims” and they have a higher risk of experiencing traumatic symptoms and adversity than “only victims” adolescents [38]. For this reason, it is important to consider preventive measures to avoid peer victimization and, thus, reduce the development of “bully-victims” adolescents. In this way, the creation of large groups of bullies that seriously damage adolescents’ HRQoL will be avoided, as well as the devastating consequences of “bully-victims” on their psychosocial well-being.

Unlike the findings of other studies [39], this research found that sociodemographic variables such as type of family or parents’ academic level do not seem to affect children’s emotional, psychological or social HRQoL domains. However, *Mood and Emotions* dimension does seem to be affected in the less privileged social classes. Furthermore, ethnic origin may affect young people’s quality of life [40], since Maghrebi adolescents and those from Latin background showed a lower quality of perceived social support. Socio-ecological interventions within adolescents’ community would be necessary to modify aggressive attitudes towards these less privileged groups. In addition, prevention and intervention efforts aiming at the less privileged social classes and non-Spanish ethnic groups should focus on creating quality social support networks to promote better HRQoL. These findings are a reminder of how important is to study HRQoL including cultural and sociodemographic factors. Thus, sociodemographic factors should also be considered to understand risk for low HRQoL.

In addition to the lack of clear evidence related to peer victimization and academic performance [20–22], there is still no research into how different types of violence independently influence academic performance. This study evidences how high levels of physical victimization are related to greater academic failure in adolescents; however, verbal/social violence seems to have no effect on academic achievement. In the same way, the analysis of the influence of the group of bullies on academic performance shows that this factor is a great obstacle to achieving academic excellence. The controversy in previous studies may be explained by the fact that victimization was study as a whole and not according to specific types of violence. This study highlights the need to draw the attention to adolescents with low academic performance and explore the possible existence of physical victimization.

The link between underprivileged sociodemographic factors and impaired academic performance is in accordance with previous research [41]. This study confirms that the most disadvantaged social classes, adolescents from non-nuclear families and those whose parents have a low educational level are at greater risk of failing academically. The same occurs with young people from non-native ethnic groups, especially adolescents from South American and Maghrebi countries, possibly due to their linkage with more disadvantaged socio-demographic factors. These results suggest the need for better academic support measures for adolescents from social classes and ethnic groups at risk of exclusion.

This study had several limitations. First, it was only analysed episodes of victimization that had taken place in the previous academic year. Such a short period of time precludes speculation about longer-term outcomes, as well as detecting relevant information about episodes of violence by peers from previous school years. Second, the cross-sectional nature of the study makes it difficult to clarify the cause-effect mechanism of the investigated associations. Future prospective studies with a longer follow-up time are necessary if data on as many numbers of violence episodes as possible is to be obtained and causal relationships between the studied elements are to be established. Third, the information was obtained from self-administered questionnaires, so the possibility of a recall bias cannot be ruled out. Cross-checking adolescents' information by different sources would be desirable in future studies. Fourth, the participation of all students was not possible, which may be related to the fact that peer victimization is linked to absenteeism [21] and some potential violence events may have gone unrecorded. Fifth, the study of cyberbullying, which is increasingly important today, was not contemplated. A study similar to the present investigation that included this phenomenon could be relevant for future research. Finally, effects of peer victimization on academic performance have been studied without controlling for academic achievement prior to victimization. It is therefore difficult to make any final conclusions regarding this finding.

On the other hand, the large sample size studied, as well as the great ethnic variety, the different social classes included and the variety of adolescent age groups, was ideal. In addition, to the best of our knowledge, this was one of the first studies to show how different HRQoL domains are affected by the type of peer victimization as well as one of the first to study the impact of the magnitude of the group of bullies as an independent risk factor for poorer HRQoL.

Taken together, this study emphasizes the harmful impact that peer victimization, in all its forms, has on the socio-psychological domains of adolescents’ HRQoL. Also, findings point to pay attention at large bullies’ groups as a factor to consider for peer victimization related interventions. The results of this study are relevant enough to continue investigating about the magnitude of the groups of bullies as an individual risk factor for poorer HRQoL. Equally, this research provides fresh reasons why school violence should continue to be regarded as a major public health problem.

## Summary

This study examines the consequences that physical and verbal/social victimization by peers and the magnitude of the groups of bullies have on academic performance and the psychological and social domains of Health-related Quality of Life (HRQoL). 1428 secondary school students in the south-east Spain completed the Spanish version of the “Adolescent Peer Relations Instrument-Bullying” and “KIDSCREEN-52” questionnaires in order to analyse, respectively, peer victimization and the psychological and social domains of HRQoL. Data on sociodemographic characteristics and academic achievement was also collected. Multivariate analyses were performed using binary logistic regression to study potential associations. Findings emphasise the potential of peer victimization in all its varieties as risk factors explaining poor HRQoL in psychological, social and emotional domains. Both physical and verbal/social victimization were strongly associated with low HRQoL in both psychological and social domains. The number of bullies was an imponent and significant risk factor explaining worse HRQoL in the five socio-psychological dimensions studied (Odds Ratio 4.08, Odds Ratio 9.25, Odds Ratio 4.69, Odds Ratio 2.91, Odds Ratio 11.92). Nevertheless, peer victimization rarely seems to affect academic achievement. Academic performance was only affected in adolescents who suffered serious physical victimization (Odds Ratio 1.67); no influence of verbal/social victimization on academic performance was detected. Groups of bullies (three bullies or more) had an independent effect on poor academic results (Odds Ratio 0.10 for attaining academic excellence). Results suggest that much of prevention and intervention are still needed to reduce peer victimization, focusing on large bullies’ groups and their harmful impact on adolescent’s HRQoL.
